# A novel autonomous real-time position method based on polarized light and geomagnetic field

**DOI:** 10.1038/srep09725

**Published:** 2015-04-08

**Authors:** Yinlong Wang, Jinkui Chu, Ran Zhang, Lu Wang, Zhiwen Wang

**Affiliations:** 1 Key Laboratory for Micro/Nano Technology and System of Liaoning Province, Dalian University of Technology, 116024 Dalian, Liaoning Province, China

## Abstract

Many animals exploit polarized light in order to calibrate their magnetic compasses for navigation. For example, some birds are equipped with biological magnetic and celestial compasses enabling them to migrate between the Western and Eastern Hemispheres. The Vikings' ability to derive true direction from polarized light is also widely accepted. However, their amazing navigational capabilities are still not completely clear. Inspired by birds' and Vikings' ancient navigational skills. Here we present a combined real-time position method based on the use of polarized light and geomagnetic field. The new method works independently of any artificial signal source with no accumulation of errors and can obtain the position and the orientation directly. The novel device simply consists of two polarized light sensors, a 3-axis compass and a computer. The field experiments demonstrate device performance.

Modern navigation technologies such as GPS have made it straightforward for mankind to locate and track global positions. These techniques are classified as satellite navigation, radio navigation, inertia navigation or celestial navigation. Unfortunately each of these techniques has their own individual weakness. For example, satellite navigation^1^ has the possibility of jamming and satellite signal loss. Radio navigation^2^ cannot work without base stations. Inertia navigation^3^ needs to correct its peculiar accumulated errors. Finally, celestial navigation^4^ is complex and can be very expensive.

Technologies derived from ancient human beings and other organisms may overcome some of the weaknesses of modern technologies. For example, Viking navigators^5^,^6^,^7^ were able to dominate the North Atlantic Ocean by using solar positioning instruments to determine the true direction. Sun-compasses were used when the sun was available. When the sun was obscured the sun position could be estimated by measuring two or more celestial points^5^ due to the polarization pattern^8^,^9^. The polarization vector is almost perpendicular to the solar vector determined by the sun and the observer. Intense study has also been carried out upon insect's eyes that often utilize polarization to navigate^10^,^11^,^12^,^13^,^14^. However, most of their activities are on small scales and their abilities are limited to orientation. Bio-polarization^15^,^16^,^17^ sensors derived from insect abilities have been constructed for orientation. The sensor consists of three polarization direction analyzers. Each polarization direction analyzer consists of a pair of POL sensors followed by a log-ratio amplifier. The information from two of three polarization direction analyzers is used to calculating the polarization angle. Long journeys can be observed in avian. Some migratory birds have position systems which can help them know where they are and which course is right for a goal^18^. In this case, both biological magnetic and celestial compasses are used as basic tools in order to help the birds to migrate. To date, these spectacular tools have not been directly imitated by humans.

Here we focus our attention on a novel global position system. The method combines the use of polarized light and geomagnetic field in order to determine the position and the orientation. This paper is organized as follows: Firstly, a novel device is constructed; secondly, the formula of the real-time position method is described in detail; thirdly, experiments are carried out.

## Methods

### Components of the position device

**Figure 1:**
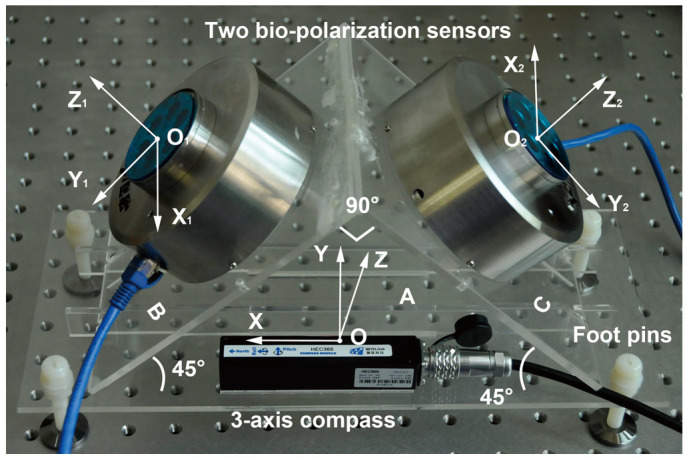
Structure of the position device and three left-handed coordinate systems. Plane A is the baseboard with a 3-aixs compass, see Supplementary Information SI for details. Plane A is made horizontal by adjusting foot pins with the help of the electronic level meter when the device is being used. Two polarized-light sensors or bio-polarization sensors are set on plane B and plane C, respectively. Three left-handed coordinate systems are built. X_1_ and Y_1_ are parallel to plane B. X_2_ and Y_2_ are parallel to plane C. The intersection lines of the three planes are parallel to each other and perpendicular to X, Y_1_ and Y_2_ respectively. The intersection angles of A and B, A and C and B and C are 45°, 45° and 90°, respectively.

A real-time position device consists of at least two polarized-light sensors, a 2-component magnetic compass, a level meter or an attitude measuring instrument and a computer which provides UTC time, an ephemerides for the sun and a geomagnetic reference model for calculation. Fig. 1 shows the main structure of our device.

### The solar angles from the cross product of two polarized-light vectors

**Figure 2:**
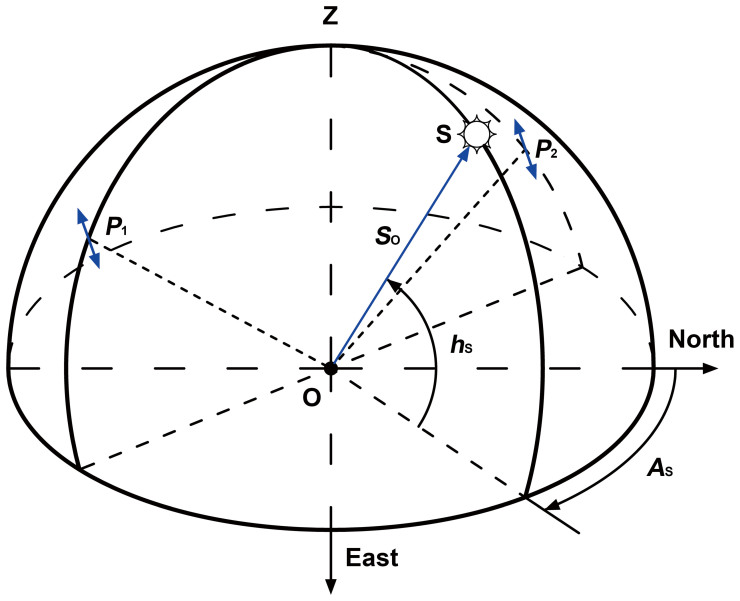
The solar vector and the polarized-light vectors. S is the position of the sun. O is the position of the observer. Z is the zenith. *A*_s_ is the solar azimuth. *h*_s_ is the solar altitude angle. The north is the geographic north. ***S*_o_** is the solar vector. ***P***_1_ and ***P***_2_ are polarized-light vectors or e-vectors. A polarized-light vector is a peculiar vector with two directions other than a standard one-directional vector. ***S*_o_**, ***P***_1_ and ***P***_2_ are all defined in the coordinate system OXYZ.

**Figure 3:**
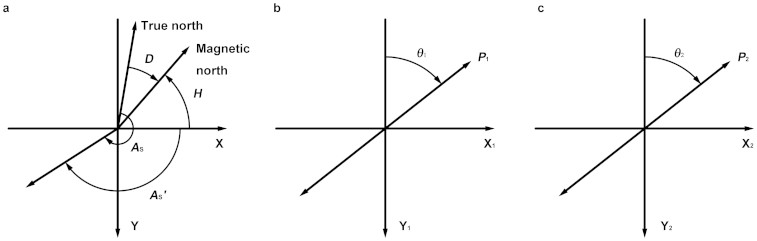
Directions of *θ*_1_, *θ*_2_, *D*, *H*, *A*_s_′ and *A*_s_ in three coordinate systems. (a) *D* is the magnetic declination. It is positive when magnetic north is east of true north or geographic north and negative when magnetic north is west of true north. *H* is the measured value of the magnetic compass. *A*_s_′ is the fake solar azimuth. (b) *θ*_1_ is the angle between the polarized-light vector ***P***_1_ and the negative direction of Y_1_. (c) *θ*_2_ is the angle between the polarized-light vector ***P***_2_ and the negative direction of Y_2_.

The solar vector can be obtained from the cross product of two polarized-light vectors measured by the polarized-light sensors. In the single-scattering Rayleigh atmosphere^19^, the direction of polarization is perpendicular to the solar vector determined by the observer and the sun as shown in Fig. 2. The solar azimuth cannot be obtained directly as the true north is unknown. Therefore, two components of the solar vector, the fake solar azimuth (Fig. 3a) and the solar altitude angle are calculated below.

The projection of ***P***_1_ in the coordinate system O_1_X_1_Y_1_Z_1_ and the projection of ***P***_2_ in the coordinate system O_2_X_2_Y_2_Z_2_ are:
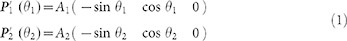
Where ***P***′_1_ and ***P****′*_2_ are the projections of the polarized-light vectors, whilst *θ*_1_ and *θ*_2_ are the measured values of the polarized-light sensors as shown in Fig. 3b–3c. Note that the coefficients *A*_1_ and *A*_2_ can take values of 1 or −1.

A movement transformation in the coordinate system is not necessary according to the property of the polarization pattern, though original points of the three coordinate systems are not coincident. By carrying out a rotation transformation in the coordinate system OXYZ we find:
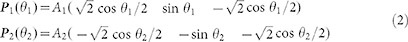


Then ***S***_o_ can be described by:
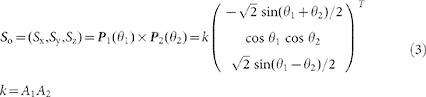
Where *S*_x_, *S*_y_ and *S*_z_ are components of ***S***_o_, *k* can take values of 1 or −1, and can be determined by naked eye judgment. The two values of *k* can be interpreted as vectors pointing opposite directions along a straight line. If *A*_s_′ and *h*_s_ are obtained when *k* is assumed to be 1, the solar azimuth and the solar altitude angle must be *A*_s_′ + 180 and −*h*_s_ respectively when *k* is assumed to be −1.

Then *A*_s_′ and *h*_s_ can be determined by:
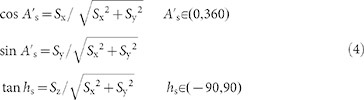


### The relationship between the solar angles and the position

From the navigation triangle built inside a sphere, we get:
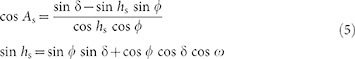
Where δ is the solar declination, *ϕ* is the geographic latitude and *ω* is the solar hour angle.

*ω* can be described by:
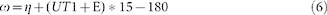
Where *η* is the geographic longitude, E is the difference between the true solar hour and mean solar hour, and *UT*1 is the local time at the 0 meridian passing through Greenwich, England. The value of *ω* ranges from −180° to +180° in theory. However it ranges from −360° to +360° in equation (6) if E is equal to 0. The periodicity of trigonometric functions means that the correct result can still be obtained.

If the measuring time (Universal Time Coordinated), which can be obtained from the computer directly is known, we can get the exact value of δ and E by inquiring the astronomical ephemeris based on the dynamical time^20^, see Supplementary Information SII for details.

### The relationship between the fake solar azimuth and the solar azimuth

By using a 2-component magnetic compass, we can obtain equation (7) as shown in Fig. 3a:
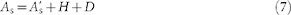


### The relationship between *D*, *ϕ* and *η*

In order to solve the problem of the parameter *D*, the IGRF (International Geomagnetic Reference Field) is used in our autonomous position method. The eleventh generation^21^ (IGRF11), which was adopted in December 2009, is a linear predictive secular variation model for 2010.0–2015.0. *D* can be calculated if *ϕ*, *η*, *r* and *UTC* are known according to the model. *r* is the geocentric altitude.

### Calculation of the position

By substituting equation (6) and equation (7) for *ω* and *A*_s_ respectively into equation (5), and ignoring the little deviation between *UT*C and *UT*1 we obtain equations (8) and (9). The downloaded program can be considered as an independent equation (10) in our method, see Supplementary Information SIII for details. Then, we get a combined equation consists of three independent equations with three unknown parameters.
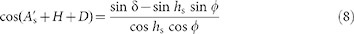

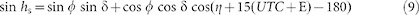

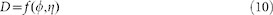


Therefore, the numerical solutions of *D*, *ϕ* and *η* can be calculated by solving the combined equation. However, there are no analytical solutions of *D*, *ϕ* and *η* in our method.

### Field experiments performed at different times

**Figure 4:**
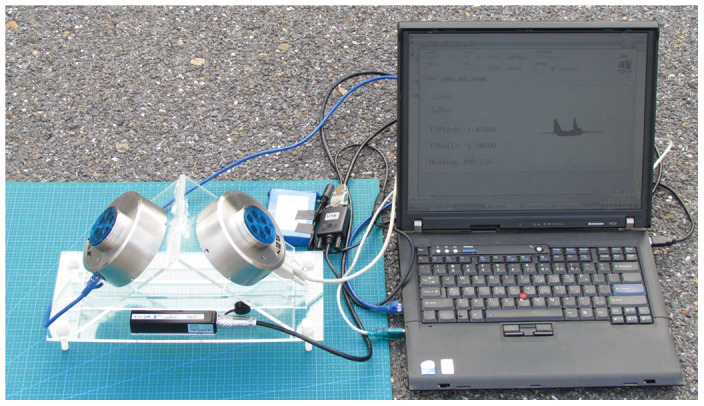
Photograph of the position device in the field. The polarized-light sensors can transmit data by using a serial port or an Ethernet port. The 3-axis compass can transmit data by using a serial port. The accuracy of the polarized-light sensor is within ±0.2° and the accuracy of the magnetic north is within ±0.5° when the angle of pitch and roll are both within ±85°.

We performed field experiments using our device (Fig. 4) in a local park (38°52′45′N, 121°31′38′E) near the School of Mechanical Engineering at Dalian University of Technology. Two sets of measurements were performed within each time period on 28 May 2014 (Beijing time) when a clear sky was available. The six time periods were before-sunrise, before-sunset, sunrise, sunset, after-sunrise and after-sunset, respectively. The initial time of each period was 19:55:48, 20:35:48, 21:15:48 on 27 May UTC and 10:25:38, 11:5:38, 11:45:38 AM on 28 May UTC, respectively. The measured value of *H* was adjusted to 92° as far as possible. The time of the second measurement was within 5 mins of the first and the value of *H* was adjusted to 272° as far as possible. Plane A was kept horizontal at all times by adjusting foot pins to keep the pitch angle and the roll angle from the 3-axis compass under 0.05°.

### Field experiments with different *H*

We performed field experiments on the roof of the building of the School of Mechanical Engineering at Dalian University of Technology on 6 Jan 2015 UTC when a clear sky was available. *H* was set to 30.6° in the first measurement and then was increased about 30° before each measurement. Twelve measurements were taken between *H* values of 30.6° to 361°. The measurement time was between 8:31:30 AM to 9:09:10 AM UTC.

## Results

### Field experiments performed at different times

**Figure 5:**
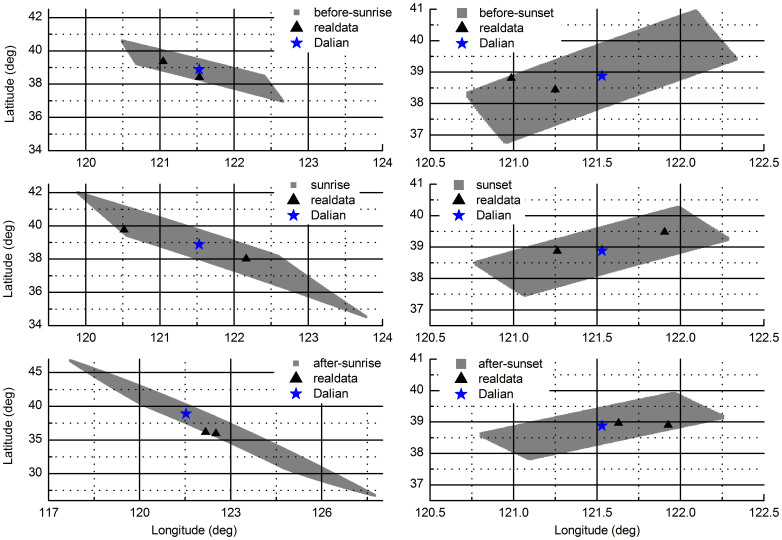
The results of field experiments at different times and error analysis in theory. ★ show the position of the park in Dalian. ▴ show the calculated position from the measured data. Generally, two solutions were obtained. ▴ were nearer to the real position of the park and considered as correct solutions. Other solutions were considered as incorrect solutions, see Supplementary Table S1 for all of the solutions. The grey areas are the sets of positions from the simulated data.

Two solutions were obtained from the combined equation at most measurement moments (Fig. 5). The k variable took the value of −1 for the first measurements and the value of 1 for the second measurements.

### Field experiments with different *H*

**Figure 6:**
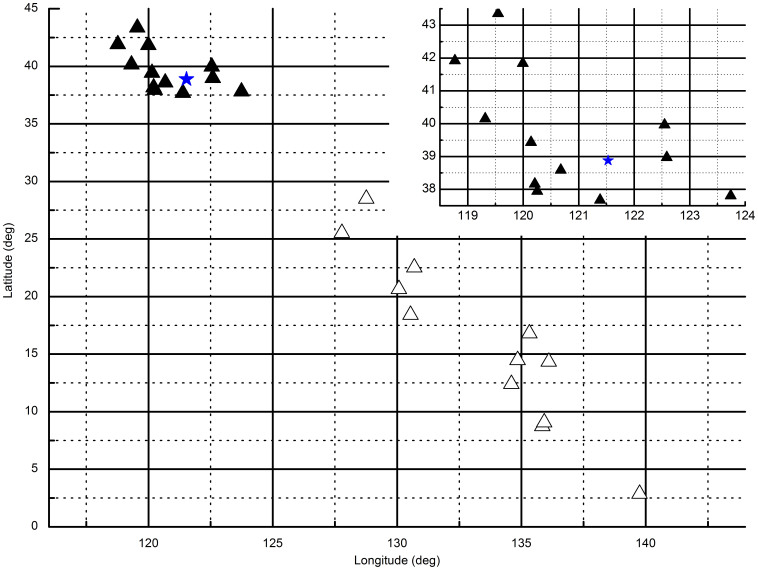
The results of field experiments with different *H*. ▴ are the solutions closer to the real position ★ and △ are the useless solutions. The area of the nearer or correct solutions has been enlarged and is placed on the top-right corner.

Two solutions were obtained from the combined equation at each measurement moment (Fig. 6). The distribution of the correct solutions was tight and the distribution of the incorrect solutions was dispersive. The *k* variable took the value of 1 for measurements between 90.2° and 240.7°. It took the value of −1 for measurements between 270° and 361° as well as from 30.6° to 60.8°. For each angular range of *H*, 6 measurements were taken. See Supplementary Table S2 for the details of *H* and *k*.

## Discussion

One or two solutions were obtained from the combined equation in most cases according to our simulation of the global positions at 10:50 AM May 28 2014 UTC and our field experiments. The cases of three or more solutions were very few. When there were more than one solution, the values of *A*_s_′ and *h*_s_ were the same and the values of *A*_s_ and *D* were different. In other words, the different valves of *A*_s_ and *D* were the main differences between the correct and incorrect solutions. We suggest that the distribution of *D* produced the wrong solutions.

It is hard to exclude incorrect or useless solutions when there is only one measurement. The vertical component of the geomagnetic field may help under this condition. However, a continuous trace exists in general when the device is in actual use. It's easy to exclude incorrect solutions in most cases as the former and the latter correct positions are very close based upon our simulation of a continuous trace. In contrast, the former and latter wrong positions were dispersive. Furthermore, the distributions of the correct and incorrect solutions were different (Fig. 6). Further research may exclude incorrect solutions by taking advantage of this property.

The set of positions obtained from the simulated data along with the random error associated with the polarized-light sensors and the compass have been calculated to discuss the impact of time on our method. The maximum error in the polarized-light sensor is 0.2°^16^. According to the datasheet, the maximum error in the magnetic north is 0.5°. The ideal value of *H* is assumed to be 92°. The time of each area is assumed to be the time of the first measurement in each period. A very accurate model of the geomagnetic field will be obtained when the device is in actual use, so the simulation has ignored the errors caused by the IGRF11. The errors caused by the pitch and roll angles have been ignored since their small errors have been merged into the error of the magnetic north. The areas from simulated data take geometry similar to a rhombus apart from the one after-sunrise that is the largest. This may be caused by the fact that two possible solutions are too close that only one solution is obtained. The three areas of the sunset periods show that the errors are smaller when the sun is going down.

Compared with a solar sensor that tests the sunlight directly, our device can work well at twilight and even when the sun is largely below the horizon. A potential advantage is the redundant information from different combinations of endless celestial points. It costs about 0.1 s to calculate a single position by solving a combined equation that only includes data measured at one moment. The computer program is running on a common computer (Computer Memory is 8 GB and CPU Clock Speed is 3.0 GHz). Therefore the measurement is essentially performed in real time.

Despite the many advantages associated with our novel measurement method, there are a number of weaknesses. The polarization pattern is not the same in all weather conditions. A research shows that migratory Savannah sparrows use polarized light cues from the region of sky near the horizon to recalibrate the magnetic compass at both sunrise and sunset^22^. The polarization pattern at sunset or sunrise is suitable for our device. The device cannot work at noon or in the night and the neutral points^8^ must be evaded. Much more work is needed to expand the working time of the device. More experiments at special places such as the poles or the equator are necessary to support the method in the further research. The polarization pattern might be changed partially by the complex geomagnetic field at the poles. What's more, the method is useless in polar nights at the poles.

*k* is a parameter that cannot be determined by the device itself. The field experiments performed at different times show that *k* points in a single direction along a straight line. The field experiments with different *H* show that the distribution of *k* may follow some rules. We suggest that the meridian of the sun is the boundary. *k* takes the value of −1 when the line OX as shown in Fig. 1 is on one side of the boundary and the value of 1 when the line OX is on the other side. The two polarized-light vectors may be parallel when the solar meridian is parallel to the axis Y. Consequently nothing useful can be obtained.

The device built by us is the simplest example of a real-time position method. Changing the spatial relationship of the three planes, the device can have many different types. By using a simple transformation of coordinates, whilst utilizing further information from the geomagnetic field, a device can be constructed without the need to adjust plane A to the local horizontal. However, the magnetic compass may have a lower accuracy if the angle of pitch or roll is large. It's obvious that the orientation can be obtained by using this paper's method without the help of the magnetic compass if the latitude and other easy-getting parameters δ are obtained. The calibration of magnetic orientation by celestial rotation^23^, which is not very clear to human beings, can tremendously simplify the method.

If the accuracies of polarized-light sensors and compasses can be improved, the accuracies of our device can be improved significantly. We are going to build better devices with higher accuracy, smaller sizes^24^,^25^ and the ability to evade the neutral points. Simple and useful ways to find the best solution from the possibilities are significant too. What's more, the simulation of accuracy performed in a global grid is also a necessary work.

## Conclusion

A novel real-time position method has been proposed that works independently of any artificial signal source and has no accumulation of errors. The algorithm has been presented in detail and a navigation device based on the method has been constructed. The experimental data indicate that the method is reliable. The method opens up new possibilities since it is a promising way to elucidate the means of avian positional navigation.

## Supplementary Material

Supplementary Information Supplementary information
