# Supplementary material for: A novel autonomous real-time position method based on polarized light and geomagnetic field
Source: Sci Rep. 2015 Apr 8;5:9725. doi: 10.1038/srep09725 (PMC4389212; doi:10.1038/srep09725)
Supplement: Supplementary Information [file srep09725-s1.pdf]

Supplementary Information:

A novel autonomous real-time position method based on polarized light and geomagnetic field

Yinlong Wang, Jinkui Chu*, Ran Zhang, Lu Wang, Zhiwen Wang

Key Laboratory for Micro/Nano Technology and System of Liaoning Province, Dalian University of Technology, 116024Dalian, Liaoning Province, China

I. The 3-axis compass

The 3-axis compass integrates a 3-axis fluxgate sensor and a 3–axis accelerometer. It can provide the angles of pitch and roll when working as an attitude measuring instrument. It can also provide the angle of the magnetic north when working as a magnetic compass. No more information can be obtained from the 3-axis compass in spite of the redundancy. All the sensors can work independently of any artificial signal source with no accumulation of errors.

II. The way to get E and δ

The dynamical time, which is used to getting E and δ by inquiring the astronomical ephemeris by using Bessel's interpolation formula, can be described:

$$TT = UT1 + \Delta T \tag{1}$$

Where TT is the dynamical time, $UT1$ is the local time at the 0 meridian passing through Greenwich, England, ΔT remains approximately constant within one year and takes the value of 67.5s in 2014 or 68s in 2015.

Considering the little deviation between UTC and $UT1$ that is under 0.9s, we have:

$$TT = UTC + \Delta T \tag{2}$$

III. The downloaded program

The open source program called "geomag70.exe" has been downloaded from the website which is

http://www.ngdc.noaa.gov/IAGA/vmod/igrf.html. It is written in C language. Before using the program based on IGRF11, three preferences had to be determined. They are the coordinate preference, the geocentric altitude (*r*) and *UTC*.

The coordinate preference of IGRF11 is set as geocentric because the navigation triangle is built inside a sphere. In our method *r* is set to 6700 km because the value of *D* from the model is substantially equal to the real one in Dalian under this condition. In IGRF11, *UTC* is accurate to the day, such as 2014-5-27. Due to this property, *D* has been calculated already before solving the combined equation. Equation (10) is considered as a database which can provide *D* when the position is known by inquiring other than calculating.

The time of 2015-1-6 on which the field experiments with different *H* were performed is slightly out of the range of IGRF11 which is 2010.0-2015.0. However, IGRF11 is still used for maintaining the consistencies of the combined equations of different field experiments.

Supplementary Table S1

All of the solutions of field experiments performed at different times

	Incorrect solutions		Correct solutions	
Time period	Lat (deg)	Lon (deg)	Lat (deg)	Lon (deg)
before-sunrise	-18.97	150.18	39.37	121.05
	-16.95	149.01	38.42	121.53
sunrise	15.35	133.90	38.02	122.16
	12.23	134.69	39.77	120.52
after-sunrise			35.95	122.52
			36.16	122.17
before-sunset	-16.73	98.13	38.44	121.25
	-16.61	97.74	38.81	120.99
sunset	-25.85	91.98	39.48	121.90
	-25.46	91.93	38.87	121.26
after-sunset	-31.88	87.38	38.97	121.63
	-31.83	86.79	39.09	121.18

Supplementary Table S2

k and *H* of field experiments with different *H*

k	*H*
-1	30.6
-1	60.8
1	90.2
1	120.4
1	150.3
1	180.1
1	210.1
1	240.7
-1	270.0
-1	301.0
-1	330.0
-1	361.0
